# Natural small molecules synergize mesenchymal stem cells for injury repair in vital organs: a comprehensive review

**DOI:** 10.1186/s13287-024-03856-4

**Published:** 2024-08-07

**Authors:** Yanling Qu, Zhe Wang, Lingjuan Dong, Dan Zhang, Fengqing Shang, Afeng Li, Yanni Gao, Qinhua Bai, Dan Liu, Xiaodong Xie, Leiguo Ming

**Affiliations:** 1 Shaanxi Zhonghong, Institute of Regenerative Medicine, Xi’an, 710003 Shaanxi Province China; 2 Stomatological Hospital, Southern Medical University, Guangzhou, 510280 China; 3 Hospital of Stomatology, Guanghua School of Stomatology, Sun Yat-sen University, Guangzhou, 510000 China; 4 School of Basic Medical Sciences, Lanzhou University, Lanzhou, 730000 Gansu Province China

**Keywords:** Mesenchymal stem cells (MSCs), Natural small molecule compounds (NSMs), Heart, Liver, Kidney, Pancreas

## Abstract

Mesenchymal stem cells (MSCs) therapy is a highly researched treatment that has the potential to promote immunomodulation and anti-inflammatory, anti-apoptotic, and antimicrobial activities. It is thought that it can enhance internal organ function, reverse tissue remodeling, and achieve significant organ repair and regeneration. However, the limited infusion, survival, and engraftment of transplanted MSCs diminish the effectiveness of MSCs-based therapy. Consequently, various preconditioning methods have emerged as strategies for enhancing the therapeutic effects of MSCs and achieving better clinical outcomes. In particular, the use of natural small molecule compounds (NSMs) as a pretreatment strategy is discussed in this narrative review, with a focus on their roles in regulating MSCs for injury repair in vital internal organs. Additionally, the discussion focuses on the future directions and challenges of transforming mesenchymal stem cell research into clinical applications.

## Introduction

The internal organs of the human body play a vital role in maintaining overall health and well-being. Injury to internal organs may lead to functional decline, loss and even life-threatening effects, so preventing injury to internal organs and promoting recovery and functional reconstruction are highly important for improving quality of life. Traditional treatment methods, such as drug therapy, surgery, intervention, laser treatment and radiotherapy, all have limitations, including inability to regenerate damaged tissues, potential complications and side effects.

**Fig. 1:**
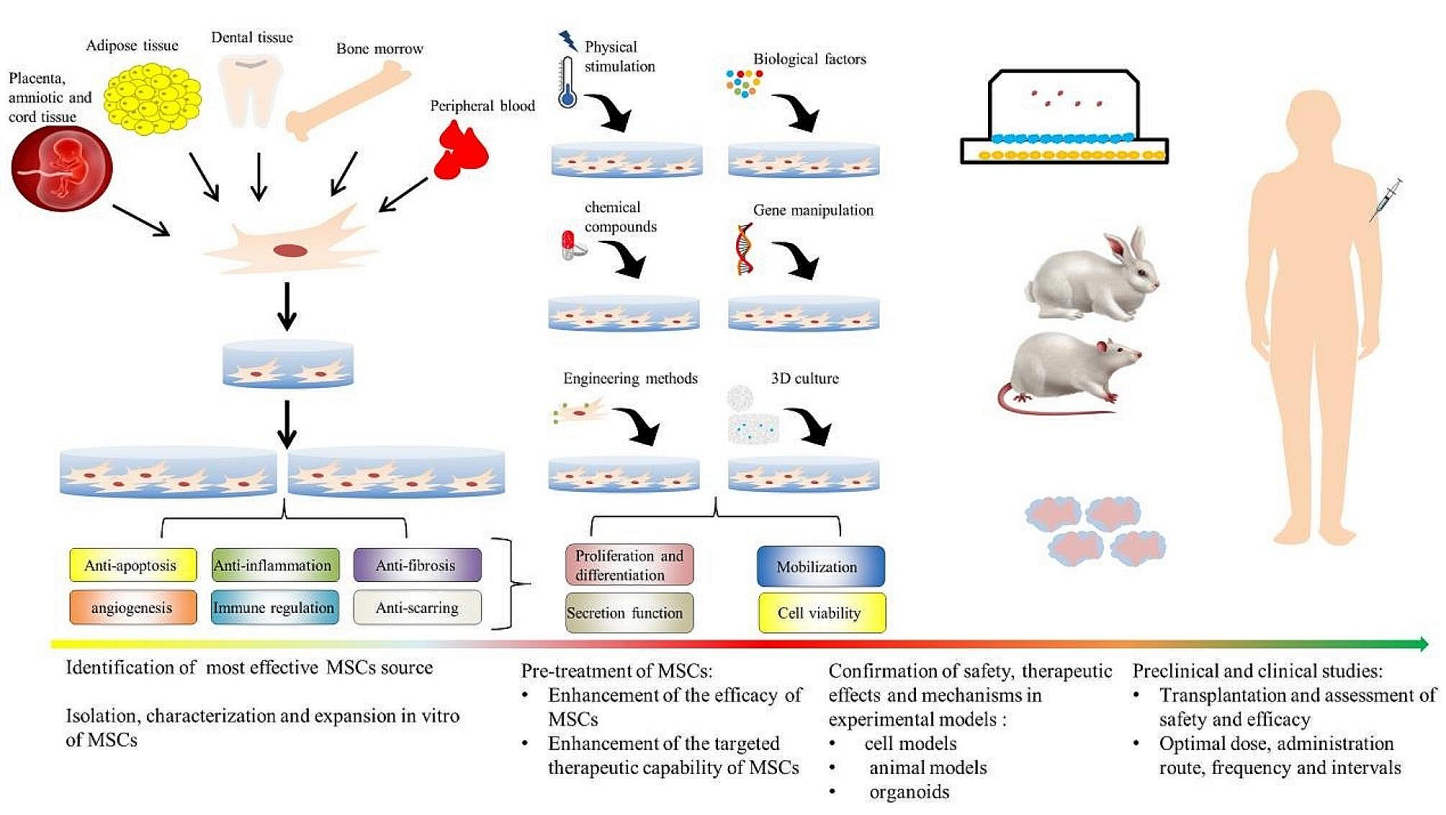
A comprehensive overview of MSCs for clinical use. Initially, the most effective source of MSCs treatment for the target disease should be determined. After MSCs are isolated from a specific source, they should be identified and appropriately expanded to ensure the quantity and quality required for treatment. Second, pretreatment strategies should be selected to enhance the therapeutic effects of MSCs. These strategies include physical stimulation (O_2_ or CO_2_ concentration, heat shock, nutrient deprivation, etc.), exposure to biological factors (cytokines/chemokines, growth factors, their combinations or biologically related samples), compounds (chemical reagents, drugs, NSMs, or other pretreatment substances), genetic engineering to manipulate the expression levels of target genes, development of a “three-dimensional (3D)” cell culture system and construction of tissue-engineered cells. The therapeutic ability of these cells should provide relevant experimental models (cell experiments and animal studies) for the evaluation of follow-up clinical research. To improve the therapeutic effect, the best conditions for the administration of MSCs, including determining the best dosage, route of administration, frequency and interval of administration, or combining them with other therapies if necessary, are needed. (Created with Microsoft PowerPoint 2010 and Adobe Photoshop CS4.)

With the continuous advancement of life science research, human understanding of stem cells has deepened gradually. Stem cell-related scientific research results have played an increasingly important role in disease treatment and regenerative medicine. Stem cell-mediated therapy is a promising alternative strategy with the potential to reduce side effects, regulate immune response and even promote tissue repair and regeneration. On the one hand, stem cells have significant applications in treating various diseases. Leukemic stem cells (LSCs) have attracted much attention in acute myeloblatsic leukemia (AML) therapy in recent years [1]. And hematopoietic stem cells (HSCs) transplantation, for example, is a common method for treating leukemia and other blood disorders. Novel treatment strategies based on stem cells, such as using induced pluripotent stem cells (iPSCs) and gene editing techniques, are providing new possibilities for treating previously incurable diseases, such as leukemia and immune system diseases, but also extend organoid technology to accelerate the development of new drugs, help precision medicine, and even advance regenerative medicine for conditions like Alzheimer’s disease and aging organ repair [2–4].Among these applications, MSCs are the most widely used. Commonly used MSCs include adipose mesenchymal stem cells (ADSCs), bone marrow mesenchymal stem cells (BMSCs) and umbilical cord mesenchymal stem cells (UCMSCs). They are characterized by their multipotential and immunomodulatory properties, which have attracted extensive attention due to their potential in tissue repair and regeneration. Nevertheless, the efficacy of MSCs transplantation in repairing internal organ injury can be limited by factors such as source availability, survival rate of MSCs as well as microenvironment influences on transplanted MSCs including inflammation, angiogenesis, cytokines, extracellular matrix components, cell-cell interactions, oxygen and nutrient availability, and metabolic environment. Moreover, preconditoning MSCs to enhance MSCs-related therapies is a promising strategy. In recent decades, various strategies have been studied to regulate the plasticity of MSCs to increase their survival, homing and therapeutic characteristics in the context of targeted diseases. These strategies include alterations in culture conditions (O_2_ or CO_2_ concentration, heat shock, nutrient deprivation, and others), exposure to inflammatory environments (cytokines/chemokines, growth factors, combinations thereof, or biologically relevant samples), pretreatment with pharmacological or other chemical molecules, and genetic engineering to manipulate the expression levels of genes of interest (Fig. 1). To enhance the curative effects of MSCs transplantation, it is common to pretreat stem cells with small molecular drugs, especially NSMs. These compounds are extracted from various plants and herbs and have demonstrated significant effects in modulating cell behavior and promoting tissue regeneration. The advantage of using NSMs in pretreating stem cells lies in their ability to better simulate the biological environment in vivo, thereby reducing the potential risks posed with manual intervention. Furthermore, these NSMs are generally more biocompatible and less toxic than other treatments currently available for use. Additionally, most NSMs discovered thus far have certain biological activities and show therapeutic effects on many diseases, such as osteoporosis, diabetes, cancer and inflammatory/autoimmune diseases [5–7]. They can simultaneously affect multiple signaling pathways, enabling more complex regulation of cell fate. On the other hand, it should not be ignored that small-molecule drugs offer an advantage because they can be fine-tuned by changing their working concentration, duration, and composition.

In particular, the ability of NSMs to regulate MSCs has garnered much attention due to their potential to enhance the repair of vital organs including the heart, liver, kidney, pancreas and other internal organs. Overall, this review aims to provide valuable insights into the role of NSMs in regulating the ability of MSCs to repair important internal organ injuries. The findings presented here are likely to promote the development of regenerative medicine and inspire future research efforts in this exciting field.

## Why MSCs

MSCs are a class of ideal cell sources for tissue regeneration owing to their excellent properties. The rich source of MSCs is the critical basis for their extensive research and applications. MSCs are known to exist in almost all tissues, including bone marrow, fat, synovium, umbilical cord, umbilical cord blood and placenta, and can be easily extracted. The multidirectional differentiation potential is another critical feature of MSCs. Different types of MSCs have different transcription groups, protein groups, immunophenotypes and immunoregulatory activities, indicating that MSCs show unique differentiation potential. They can be differentiated into different cell lineages based on the requirements of specific biomedical applications. Furthermore, the differentiation tendency and proliferation capability of MSCs are influenced by different tissue sources. There are an increasing number of publications addressing the heterogeneity of MSCs [8]. Moreover, because MSCs do not express significant histocompatibility complexes or immunostimulatory molecules, they cannot be detected by immune monitoring, nor will they lead to graft rejection after transplantation [9, 10]. These characteristics make them potential candidates for biomedicine, especially in the field of tissue engineering.

On the other hand, MSCs are capable of homing to injured sites and releasing chemokines, cytokines, and growth factors that aid in tissue regeneration [11]. All in all, many advantages of MSCs such as their self-renewal, in vitro proliferation, rapid cell doubling capacity, anti-fibrotic, anti-apoptotic, anti-inflammatory, immunomodulatory and immunosuppressive effects, and also paracrine nature have been demonstrated in various pre-clinical studies and clinical evidence. In view of these characteristics, in animal models and human clinical trials, MSCs have shown promising results for repairing damaged tissues in various diseases, including diseases affecting the heart, liver, kidney, and pancreas.

## Classification of NSMs for MSCs-related therapies

NSMs are monomeric compounds with molecular weights less than 1000 kDa that are extracted from natural plants, animals, microbes or minerals. In recent years, NSMs have been widely used in the food, pharmaceutical and cosmetic industries because of their rich sources, complex and diverse structures and extensive and unique biological activities. An increasing number of studies have shown that NSMs, as tools, can accurately regulate the cell fate of MSCs, so a great deal of research has been conducted on the additional benefits and applications of NSMs in disease treatment and tissue regeneration. Some well-known ingredients (such as allicin, hesperidin and artemisinin), derived from food components, plants or Chinese herbal medicine, respectively, are widely studied as effective NSMs due to their pharmacological activities (such as anti-inflammatory, antioxidant and antiviral effects) [12]. At present, the use of NSMs, such as saccharides, glycosides, phenylpropanoids, quinones, flavonoids, terpenoids, volatile oils, steroids, and alkaloids, to control MSCs may provide potential solutions to overcome many limitations and further maximize the effectiveness of MSCs-related applications. Two aspects of the ability of these NSMs to amplify the therapeutic effects of MSCs-related applications have been investigated: (1) the engraftment, survival and differentiation of MSCs and (2) the amelioration of the MSCs microenvironment.

### Survival and differentiation of MSCs

MSCs undergo senescence upon repetitive passaging in vitro, and a lower survival rate in vivo limits their therapeutic value for clinical applications. Several studies have proven the effects of NSMs on the survival and differentiation of MSCs both in vivo and in vitro. Moreover, many NSMs have been shown to prevent aging in MSCs and further promote the proliferation of MSCs. Studies have also shown that MSCs differentiate into osteoblasts, nerve cells and endothelial/vascular progenitor cells in the presence of NSMs.

Polysaccharides are the main NSMs in carbohydrates and can regulate the proliferation and differentiation of MSCs. Astragalus polysaccharide (APS) is extracted and isolated from the roots and stems of *Astragalus membranaceus*, a leguminous plant. APS promoted the osteogenic differentiation of BMSCs by facilitating ankyrin repeat and FYVE domain containing 1 (ANKFY1) expression through miR-760 inhibition [13] and promoted the proliferation and adipogenic differentiation of BMSCs cultured in a hypoxic environment [14]. Polygonatum sibiricum polysaccharide (PSP) is also a traditional Chinese medicine that has been widely used to treat many diseases for hundreds of years. During the process of osteogenic differentiation, PSP also promoted the proliferation of BMSCs and enhanced their viability [15]. Liao et al. [16] evaluated the effect of Angelica sinensis polysaccharide (ASP) on the osteogenic differentiation of BMSCs in high-glucose (HG) rats. ASP can promote the osteogenic differentiation of rat BMSCs (rBMSCs) under high glucose conditions and induce bone regeneration in type 2 diabetic rats, possibly through the activation of the Wnt/β-catenin signaling pathway. Curculigoside is a natural phenolic glycoside compound produced by *Curculigo orchioides Gaertn*. Curculigoside can also induce the osteogenic differentiation of ADSCs and prevent bone loss in an ovariectomy (OVX) model through the phosphoinositide 3-kinase (PI3 K)/protein kinase B (AKT) signaling pathway [17]. *Ajuga decumbens* extracts were shown to stimulate the osteogenesis of MSCs [18]. Honokiol improved the chondrogenesis of MSCs [19]. However, Achyranthes bidentata polysaccharides (ABPS) can inhibit the adipogenic differentiation of BMSCs, and this mechanism may be related to the regulation of the peroxisome proliferator activated receptor gamma/ transient receptor potential vanilloid 4 (PPARγ/TRPV4) pathway [20]. *Cornus walteri* extracts, *Oryza sativa* extracts, and piceatannol (enriched in *Aiphanes horrida*) were also shown to inhibit the adipogenesis of MSCs [21–23].

*Dendrobium officinale* is an important traditional Chinese medicine, and its main component, polysaccharides, has many pharmacological activities. Dendrobium officinale polysaccharide (DOP) promoted the neuronal differentiation of BMSCs by increasing the expression levels of neuron markers [24]. In addition, in the process of DOP-induced differentiation of BMSCs into neurons, the Notch signaling pathway was inhibited, and the Wnt signaling pathway was activated.

Resveratrol (RSV) is a nonflavonoid polyphenol phytoalexin extracted from multiple plants, including the roots of white hellebore (Veratrum grandiflorum). RSV has been proven to be involved in regulating chondrogenesis and neuronal differentiation of MSCs by regulating different signaling pathways [25–29]. Erucic acid, a new natural component of *rosemary*, regulates BMSC differentiation by inhibiting PPARγ transcription. Furthermore, 25 µM erucic acid significantly decreased the expression of adipocyte marker genes while accelerating the expression of osteoblast marker genes [30].

Phenylpropanoids are naturally occurring phenolic substances with phenol structures. For the first time, Moustata et al. revealed that eugenol (EUG) significantly improved the self-renewal, migration and proliferation of ADSCs in vitro [31]. The effects of cinnamaldehyde (CA) and EUG on the viability, doubling time and adipogenic or osteogenic differentiation of ADSCs were also investigated. CA and EUG, used at low concentrations, strongly enhanced the osteogenesis and decreased the adipogenesis of ADSCs [32]. The administration of fucoidan-pretreated MSCs has been shown to improve MSC function through senescence inhibition, increased angiogenesis, and enhanced cell proliferation [33].

Furthermore, naringin, a flavonoid compound that is commonly found in citrus fruits and the traditional Chinese medicine *Rhizoma Drynariae*, reversed the decreases in osteogenic gene expression (Runx2, Osx) and ALP activity induced by tumor necrosis factor -alpha (TNF-α) or hydrogen peroxide [34, 35].

Generally, the discovery of new NSMs has changed the fate of MSCs, including their survival and differentiation. The regulation of NSMs is multifaceted and governed by multiple signaling pathways, epigenetic regulation, and posttranslational modification. In addition to the abovementioned small molecules, many other recently discovered small molecules that affect the proliferation and lineage orientation of MSCs deserve further research.

### The amelioration of the microenvironment

Many NSMs show remarkable antioxidant and anti-inflammatory effects and can adjust the microenvironment of MSCs. The behaviors of MSCs are affected by their microenvironment in situ, and the underlying mechanisms are mainly related to promoting the survival, differentiation and secretion of MSCs by reducing oxidative or endoplasmic reticulum stress, reducing inflammatory cytokines, inhibiting the infiltration of immune cells, reducing the expression of apoptotic messengers and enhancing the expression of supporting contributors [36].

Osthol is a natural pyrogen that was originally extracted from *Fructus Cnidii* and has been proven to enhance the curative effect of BMSCs therapy. Injection of osthol-treated BMSCs restored the immunosuppressive ability of BMSCs, thus reducing lymphocyte infiltration and mucosal damage [37]. Astragaloside is a pure compound extracted from *Astragalus*. Combined treatment with baicalein can inhibit the expression of interleukin (IL)-1β, IL-8, and TNF-α in lipopolysaccharide (LPS)-stimulated BMSCs [38].

APS upregulates the proteins B-cell lymphoma-2 (Bcl-2) and Bcl-xl and downregulates the proteins Bax and Bak, which leads to a decrease in the mitochondrial membrane potential (MMP), thus inducing the release of cytochrome C (Cyto-c) and apoptosis inducing factor (AIF), which leads to caspase-dependent and caspase-independent pathways leading to apoptosis [39]. These results indicate that APS can inhibit the accumulation of mitochondrial reactive oxygen species (ROS), thus inhibiting the activation of the mitochondrial pathway and further preventing the cell damage caused by X-ray radiation-induced bystander effects (RIBE) on BMSCs [40]. According to the findings of Zhang et al., APS blocks RIBE by regulating the MAPK/NF-κB pathway [41]. Moreover, treatment of BMSCs with APS to inhibit mitochondrial ROS accumulation can markedly inhibit apoptosis, senescence, and the reduction in proliferation and pluripotency caused by ferric ammonium citrate (FAC)-induced iron overload [42]. APS can protect against the cytotoxicity and genotoxicity of formaldehyde (FA)-induced effects on BMSCs. The mechanism may be related to the upregulated expression of xeroderma pigmentosum group A (XPA), xeroderma pigmentosum group C (XPC), excision repair cross-complementation group 1 (ERCC1), replication protein A1 (RPA1), and replication protein A2 (RPA2) in the nucleotide excision repair (NER) pathway, which promotes DNA damage repair [42].

On the other hand, the activation of autophagy was demonstrated to be positively correlated with apoptosis. Berberine (BBR), a main extract from *Coptis* and *Phellodendron*, reduced ROS levels and regulated apoptotic proteins and thus alleviated ADSC apoptosis under hypoxic and serum-deprived conditions. BBR reversed the increase in p-AMPK and decrease in p-mTOR induced by starvation, which indicated that BBR could alleviate autophagy by regulating the AMPK-movement pathway and promoting cell survival [43]. In addition, the lysosome plays a crucial role as a degradation site in the process of autophagy. Lysosome acidification is essential for its normal function, and any dysfunction in this acidification process can result in aging and death of MSCs [44]. Vacuolar H+-ATPases (V-ATPases) and lysosomal ion channels and transport proteins play a crucial role in the process of lysosomal acidification. Supplementation with baicalein increased the expression of V-ATPase V1 subunit and co-localization of V1 subunit in mouse lysosomes via the mTOR pathway [45]. Nie et al. also found that Dendrobium nobile Lindl. alkaloids (DN LA) could increase the expression of the A1 subunit of v-ATPase to promote lysosomal acidification [46]. Additionally, Dehydropachymic acid (DPA) treatment restored the bafilomycin A1-induced increase in lysosomal pH [47].

Furthermore, long-term exposure to HG microenvironment in vitro and in vivo is harmful to cells and has physiological and pathological consequences. HG causes changes in stem cell biology, affecting their normal behavior and function during tissue repair, thus interfering with the quality and efficacy of stem cells [48]. There is a possibility of immunomodulatory shift in MSCs after being cultured in HG environment, and HG impairs mitochondrial dynamics as well as regulatory proteins leading to poor survival rates and high apoptosis rates under this condition [49, 50]. Research has shown that RSV enhances MSC viability under HG environment by reducing miR-34a expression [51, 52].

## NSMs as complementary regulators of injury repair by MSCs

Due to the above-described characteristics, it is no surprise that there is great interest in researching the potential of NSMs for the therapeutic use of MSCs. In various studies, NSMs and MSCs have been explored for treating different diseases and damaged tissues. The next section will summarize the research results that support the potential use of MSCs in the treatment of major organ systems, including the repair and regeneration of the cardiovascular system, liver, kidney, pancreas and other internal organs.

### Cardiac disease and injury

Cardiovascular diseases (CVDs) are the leading cause of death worldwide. It is estimated that by 2030, approximately 24 million people will die from CVDs [53]. Many pathological processes in the heart eventually lead to myocardial cell damage, and long-term decreases in myocardial contractility. These conditions are usually accompanied by the development of heart failure (HF), which is the main cause of death and morbidity in developed countries. Among patients with diabetes mellitus (DM), the risk of HF is 2 to 5 times greater than that in patients without DM [55]. It is predicted that by 2025, the prevalence of diabetes will increase to 5.4%, and diabetes will affect approximately 300 million people worldwide. The large health and economic burden of this disease also urges us to take measures to alleviate heart damage in DM patients. MSCs therapy has attracted widespread attention for its ability to promote heart repair and cardiac function recovery. NSMs have been used to promote the survival, proliferation and homing of MSCs under stress conditions such as ischemia, hyperglycemia or aging (Table 1, Additional file 1).

#### Cardiac differentiation

There are many NSMs have been demonstrated to enhance the differentiation of stem cells into myocardial cells, offering potential treatment for cardiac diseases. Monika et al. showed that retinoic acid (RA), EGCG, and vitamin C (VitC) alone and combinations of RA, EGCG, and VitC with angiotensin II were able to induce alterations in human amniotic fluid-derived mesenchymal stem cells (AF-MSCs) at the phenotypic, genetic, protein, metabolic, and epigenetic levels, leading to the formation of cardiomyocyte progenitors that may become functional heart cells in vitro or in vivo [56]. MSCs were induced to differentiate into endothelial cell (EC)-like cells in vitro. Tanshinone IIA (Tan IIA) and Astragaloside IV (AGS-IV) may promote the angiogenesis of EC-like cells by upregulating the expression of connexin 37 (Cx37), Cx40 and Cx43 and enhancing gap junctional intercellular communication (GJIC) function [57]. These results may provide experimental evidence that these two compounds play synergistic roles in the treatment of coronary heart disease (CHD) with MSCs. In addition, Akbar et al. suggested that the combination of valproic acid and 5-azacytidine with ascorbic acid (AA) and salvianolic acid B (Sal B) can promote the cardiac differentiation of MSCs [58]. Moreover, the hypothesis that bone morphogenetic protein-2 (BMP-2) combined with Sal B can promote the differentiation of rBMSCs into cardiomyocytes (CMCs) in vitro was verified by Lv et al. [59].

#### Myocardial infarction (MI)

Guo et al. further proposed that Sal B alone may play a synergetic role in the cytoprotection of BMSCs and that Sal B-BMSCs transplantation can significantly improve the heart function of rats with MI by enhancing angiogenesis and the production of beneficial cytokines, such as vascular endothelial growth factor (VEGF) and angiopoietin 1 (Ang-1) [60].

High levels of ROS and free radical damage are involved in the senescence and apoptosis of MSCs, leading to neovascularization and tissue repair defects. Therefore, it is difficult for MSCs to exert their maximum therapeutic effect under ischemic conditions. Lycopene is a carotenoid that occurs naturally in tomato and tomato plant extracts and has strong free radical scavenging activity. Lycopene can inhibit the production of ROS through the PI3K/Akt-MnSOD pathway, thus protecting MSCs from ischemia-induced apoptosis, which indicates that lycopene could be developed as a useful broad-spectrum reagent for successful MSC transplantation [61]. Han et al. [62] evaluated the role of melatonin (MT) in the cardiac protection of ADSCs. They found that MT promoted the survival of transplanted ADSCs in vivo and improved cardiac function after MI injury. They further clarified the mechanism by which MT inhibits excessive inflammation and oxidative stress through a SIRT-dependent mechanism. Liu et al. [63] investigated the effects of Ginkgo biloba extract (EGb) 761 on the cardiac performance of infarcted hearts by increasing the viability of implanted BMSCs. EGb 761 protected the implanted MSCs from MI-induced apoptosis by activating Fas-mediated death receptor signaling pathways. Furthermore, prevention of inflammation, apoptosis and oxidative stress enhanced the survival and differentiation of the transplanted MSCs.

Rapamycin was first isolated from *Streptomyces hygroscopicus*, a soil bacterium, in 1975, and it has effective antifungal and immunosuppressive properties. Rapamycin can significantly increase the autophagy activity of MSCs, reduce apoptosis and promote the secretion of various growth factors [64]. Therefore, transient pretreatment with rapamycin promoted the survival and differentiation of engrafted MSCs in ischemia/reperfusion (I/R) myocardium. After engraftment of rapamycin-pretreated MSCs, repair of the infarcted myocardium and restoration of cardiac function increased dramatically.

#### Diabetic cardiomyopathy (DCM)

Hyperglycemia impairs cell viability and induces cell senescence, so it is difficult for MSCs transplantation alone to play a role in the treatment of DCM under high glucose conditions. Ginsenoside RG1 (RG1)-induced MSCs secrete exosomal circNOTCH1 and alleviate DCM by activating the NOTCH signaling pathway to induce macrophage M2 polarization [65]. Many studies have shown that RSV enhances the effect of MSCs in the treatment of DCM through different signaling pathways. Zhang et al. [51] reported that RSV preconditioning reduced the expression of miR-34a in BMSCs stimulated with HG and rejuvenated BMSCs under hyperglycemic conditions. Further analysis revealed that RSV-based transplantation promoted cardiac recovery after infarction in diabetic rats, promoted the release of angiogenic factors and increased the density of arterioles and capillaries. RSV-preconditioned BMSCs can also improve cardiac remodeling via the downstream Wnt/β-catenin pathway and attenuate sFRP2-mediated fibrosis and further promote angiogenesis [66]. In addition, RSV pretreatment can improve the survival of ADSCs in a HG environment and inhibit cell apoptosis, and antioxidant stress is enhanced by upregulating the Sirt1, PGC1α and p-Akt pathways. The migration rate and paracrine effect of adipose-derived stem cells are increased by increasing the expression of CXC chemokine receptor4 (CXCR4), thereby improving the cardiac function of DCM patients [67]. NSMs pretreatment can not only enhance the function of MSCs but also increase the extent of repair caused by the oral administration of NSMs when MSCs are transplanted. Chen et al. [68] found that oral administration of the green tea EGCG can reduce oxidative stress and enhance the restoration of cardiac function in diabetic rats receiving autologous transplantation of ADSCs. Oral EGCG administration synergistically enhances cardiac function in DM rats treated with MSCs [69]. The cross effect of EGCG and BMSCs therapy occurs through the expression of survival proteins.

#### Age-associated cardiac disorders

The increasing aging population has increased the challenges of age-associated cardiac disorders. *Alpinate Oxyphyllae Fructus* (Alpinia oxyphylla Miq, AOF) is a well-known Chinese medicine that has been demonstrated to possess both anti-aging effects and cardioprotective effects [70]. Chang et al. reported that retreating ADSCs with AOF extract can enhance the expression of stem cell markers, increase the levels of longevity-related proteins such as Sirt1, and increase the migration of damaged cardiac cells [71]. AOF-pretreated ADMSCs may activate paracrine factors that have been demonstrated to have a protective effect against senescence in cardiac cells by suppressing NF-κB signaling and enhancing survival signaling. In addition, they discovered that AOF-pretreated ADMSCs alleviate mitochondria-mediated cardiac apoptosis, increase DNA replication and decrease S phase-mediated DNA replication in senescence-associatedCMCs, which reduces senescence-associated apoptosis [72]. The combined treatment of AOF with ADSCs helps in overcoming aging-associated cardiac apoptosis.

### Liver disease and injury

The morbidity and mortality of liver disease are very high, and liver disease is the main threat to human health [73]. Many stimulating factors, such as viral hepatitis, alcoholism, drugs, metabolic diseases and autoimmune attack, can cause chronic/acute liver injury and inflammation, leading to liver failure, liver cirrhosis and related hepatocellular carcinoma. The results are shown in Table 2 (Additional file 2).

#### Inflammatory liver disease

Hepatic inflammation is the basic cause of liver diseases. According to research, acute graft-versus-host disease (aGVHD) is an inflammatory disease that can damage all parts of the body, thus causing death. Asarin is derived from the leaves of the *Lauraceae*. Asarinin-pretreated UCMSCs can significantly inhibit the proliferation of CD4 + and CD8 + T cells and reduce inflammatory damage to the liver in an aGVHD mouse model. In addition, asarinin can cooperate with IFN-γ to promote the secretion of indoleamine 2,3-dioxygenase by UCMSCs and induce allograft tolerance [74]. Inflammatory responses also contribute to nonalcoholic fatty liver disease (NAFLD). However, there is currently no effective treatment for NAFLD, which increases the risk of developing nonalcoholic steatohepatitis (NASH), cirrhosis, or liver cancer [75].

Long-term chronic inflammation in the liver leads to liver fibrosis, which can ultimately progress to end-stage liver diseases such as liver cirrhosis and liver failure. Hepatic fibrosis is caused by hepatocyte injury, which leads to liver structure distortion and loss of liver function [77]. Zhang et al. reported that both MSCs and ferulic acid (Fa) can effectively alleviate liver fibrosis. Combined treatment was more effective than treatment with Fa or BMSCs alone because of the inhibition of cytoskeleton rearrangement and the transfer of miR-19b-3p to activated hematopoietic stem cells (HSCs), which inactivates the RhoA/ROCK signal [78]. Fathy et al. also reported that combined treatment with EUG and ADSCs was more effective than single-component therapy and strongly inhibited the progression of CCl4-induced hepatic fibrosis through inhibition of the transforming growth factor β (TGF-β)/Smad pathway [79]. Baig et al. showed that vitamin E (VitE) pretreatment can boost the antifibrotic effects of Wharton’s jelly derived MSCs (WJMSCs). VitE, a lipophilic antioxidant, plays an important role in the redox homeostasis of cells. VitE-WJMSCs transplantation significantly improved the structure and functions of the liver, leading to the cessation of fibrosis [80]. Mortezaee et al. noted that pretreatment of MSCs with MT had a better ability to home to damaged livers and to balance matrix degradation and accumulation factors, so it had a better therapeutic effect on animal models of liver fibrosis [81].

#### Acute liver failure (ALF)

ALF is a life-threatening disease. Icariin (ICA) is an active component of *Epimedium* and has the potential to promote the proliferation of MSCs. MSCs alone increased the survival rate of ALF rats and reduced liver damage, while MSCs cocultured with ICA exerted a greater therapeutic effect. ICA improved the antiapoptotic potential of MSCs by upregulating the hepatocyte growth factor (HGF)/c-Met pathway [82]. In addition, Lai et al. demonstrated that L-theanine-pretreated ADSCs have antitumorigenic effects on a liver injury rat model. L-theanine enhances the therapeutic effects of ADSCs by promoting the secretion of HGF, thus improving the healing of damaged liver tissue [73]. Doxorubicin (DOX), one of the most effective chemotherapeutics against cancer, is reported to accumulate in patients with liver cancer due to its detoxification capacity [83]. Pretreatment of BMSCs with hyaluronic acid (HA) for 14 days increased the proliferation of MSCs and CD44 and SDF1α gene expression, indicating that HA treatment enhances cell viability, proliferation, and cell migration. Compared to BMSCs alone, HA-pretreated BMSCs led to considerable improvements in liver function and morphology via Wnt/β-catenin pathway inhibition in an acute liver damage model [84]. Moreover, the expression of inflammatory markers (TGFβ1 and iNOS), apoptotic markers (Bax and Bcl2), cell tracking markers (SDF1α), fibrotic markers (β-catenin, Wnt7b, FN1, VEGF, and Col-1), and ROS markers (Nrf2 and HO-1) increased greatly to near-normal levels.

#### Hepatic resection and regeneration

How to regenerate the retained liver and restore its structure and function after different proportions of hepatectomy due to trauma, infection, metabolism and poisoning is an important subject in clinical and laboratory research. RSV and MSCs alone or together reduce the levels of TNF-α and IL-6 while increasing the levels of HGF [85]. The coadministration of RSV and MSCs increases the number of live MSCs in the livers of rats subjected to partial hepatectomy (PH). This may be related to the life-prolonging effect of RSV on MSCs, which leads to enhanced liver regeneration. Hepatic I/R injury is a complex pathophysiological process that can lead to initial poor or primary dysfunction of liver function and increase morbidity and mortality after hepatectomy and liver transplantation. Rapamycin preconditioning can enhance the therapeutic effect of hUCMSCs on liver I/R injury by inhibiting liver inflammation and oxidative stress [86]. The results of in vitro studies further showed that rapamycin can induce autophagy in MSCs by upregulating the expression of the CXCR4/CXCL12 axis, enhancing their migration ability and promoting their homing to damaged liver tissue.

#### Diabetic liver disease

In addition, previous studies have confirmed that the function of stem cells is reduced in high-sugar environments, which impairs their regenerative ability. However, Chen et al. noted that RSV pretreatment increased the viability of ADSCs under high glucose stress through the expression of Sirt1 and IGF1R, and the secretion of IGF1 in ADSCs pretreated with RSV also increased, therefore, RSV may be used to treat liver dysfunction in DM patients with liver dysfunction [87].

### Kidney disease and injury

Kidney disease is a global public health problem that affects more than 750 million people worldwide and causes 5 million to 10 million deaths every year. The main common diseases are acute kidney disease (AKD) and chronic kidney disease (CKD) [55]. MSCs-based therapies have been widely studied for the treatment of kidney disease, and they have been shown to improve renal function and restore damaged renal tissue in animal research and clinical trials [88]. In the treatment of kidney disease, some NSMs have been proven to have protective effects on enhancing the survival and proliferation of MSCs, as shown in Table 3 (Additional file 3).

#### AKD

AKD is a common clinical syndrome characterized by a sudden loss of renal function. MSCs play a role in relieving AKD through a variety of mechanisms [89]. Based on the various biological functions of RSV, such as its anti-inflammatory, anti-oxidative, and anti-aging effects, it has been widely studied in regenerative medicine. RSV-hUCMSCs secrete more platelet-derived growth factor (PDGF)-DD into renal tubular cells, which activates the extracellular signal regulated kinase (ERK) pathway, inhibits renal tubular cell apoptosis, enhances the proliferation of hUCMSCs, and promotes angiogenesis in endothelial cells; eventually, RSV-hUCMSCs play a more effective repair role than hUCMSCs in AKD [90]. *Asian pigeonwing* plants (*clitoria ternatea*) contain many effective components, such as flavonoids, phenols and anthocyanins, and have many pharmacological activities, such as antibacterial, anti-inflammatory, antioxidant, antitumor and antifibrotic effects. Combined treatment with *Asian pigeonwing* extracts and MSCs restored the standard functions of cisplatin-treated rats. This combination strategy restored the SOD and GSH levels somewhat toward normal, and the results indicated its potent lowering effect on the parameters measured, which were creatinine, uric acid, and urea [91]. However, further detailed research is needed to fully understand the actual mechanism of the interaction between stem cells and plant extracts. Whether other forms of AKD or larger sample sizes would produce the same results remains to be discussed. The combination of VitE and UCMSCs plays a favorable role in regulating the levels of inflammatory cytokines in AKD mice, which have the best renal function and least renal tubular damage [92].

#### CKD

CKD, which is characterized by a low glomerular filtration rate, proteinuria, high morbidity and mortality, is characterized by damage to kidney structure and function. ICA-treated BMSCs alleviate oxidative damage and inflammatory reactions and promote the expression of growth factors through paracrine action, thus accelerating the therapeutic effect on CKD [93]. Chitin powder is a natural biopolymer that is mainly extracted from the exoskeleton of crustaceans. ADSCs coculture with chitin powder (C-ADSCs) significantly increased the production of HGF and VEGF, and C-ADSCs injection reduced proteinuria and significantly increased the expression of podocin, which indicated that damage caused by podocin was alleviated in a rat model of CKD induced by DOX [94]. These results may provide a new strategy for the treatment of CKD. However, it is not clear whether factors other than VEGF and HGF play a role in this process, and whether the injected cell dose affects the treatment effect needs more research. HA is another kind of natural polysaccharide that is dispersed in many tissues. It seems that the renal protection of BMSCs treated with HA is mainly due to the inhibition of inflammation and fibrosis. Sreag et al. [95] reported that pretreatment of BMSCs with HA decreased the expression of the apoptosis marker Caspase-3, the inflammatory markers TNF and IL-6, and the fibrosis markers Wnt7a, β-catenin and fibronectin 1, and that HA may play a key role in inhibiting the Wnt/β-catenin pathway. However, this research has several limitations, such as a lack of long-term follow-up of BMSCs, lack of safety assessment, and unclear sources of HA. These limitations need to be further studied to enhance the scientific rigor and persuasiveness of the research.

MT has been found to be an effective free radical scavenger through its antioxidant and anti-inflammatory properties [96], and it plays a therapeutic and protective role in kidney diseases (including AKD and CKD) through different mechanisms [97]. For example, Chen et al. showed that treatment of apoptotic ADSCs (a-ADSCs) with MT can play an additional role in alleviating sepsis-induced AKD. The combined therapy had better therapeutic effects on anti-inflammatory, antioxidation, antiapoptotic and antifibrotic effects and on the circulating creatinine level [98]. Hematoxylin and eosin (HE) staining also confirmed less kidney injury in the combined treatment group. In vitro and in vivo experiments further verified these results. Mias et al. transplanted BMSCs preconditioned with MT into I/R-induced AKD (I/R-AKD) rats. In the pretreatment group, more surviving transplanted MSCs, excessive stimulation of angiogenesis and proliferation in renal cells and faster recovery of renal function were observed, which indicated the potential role of MT in protecting transplanted MSCs in the in vivo microenvironment [99]. In vitro analysis revealed that MT could protect MSCs from apoptosis and promote angiogenesis/mitogen secretion. Saberi et al. [100] reported that more engrafted and surviving MSCs preincubated with MT were observed in the injured kidneys of unilateral ureteral obstruction (UUO) rats, suggesting better survival and migratory activity of MSCs. Diabetic nephropathy (DN) is the main cause of CKD. Rashed et al. also reported that MT pretreatment significantly enhanced the proliferation of MSCs in vitro. Compared with those in the control group, the DN rats that received MT-MSCs exhibited improved renal function, reduced inflammation and fibrosis, and increased levels of antioxidant enzymes and the autophagy protein Beclin-1, all of which indicated that pretreatment of MSCs with MT further enhanced their therapeutic effects [101].

### Pancreatic disease and injury

The pancreas is an important organ in the human body. Diabetes and pancreatitis are two common pancreatic diseases. Studies assessing the efficacy of MSCs preconditioning with NSMs for pancreatic disease and injury are shown in Table 4 (Additional file 4).

#### Diabetes

DM characterized by hyperglycemia is a type of pancreatic injury. RSV has been proven to have effective antidiabetic, antioxidative and anti-inflammatory effects, and it is a promising adjuvant drug for the treatment of diabetes based on MSCs. Cell experiments have shown that RSV can revive MSCs under hyperglycemic conditions [51, 102]. RSV has a protective effect on the senescence of MSCs and can preserve the paracrine effect of MSCs on promoting insulin secretion by INS-1 (rat islet cell tumor cell) cells via Pim-1 [103]. A previous study revealed that autologous transplantation of MSCs combined with oral administration of RSV or RSV-pretreated MSCs has a protective effect on damaged pancreatic tissues in DM rats [102, 104–106]. Pretreatment of MSCs with RSV in vitro increases the expression of the survival marker p-Akt, leading to enhanced ADSCs viability and significantly improving islet function in diabetic rats [102]. The autologous transplantation of ADSCs coupled with the oral administration of RSV also significantly improved pancreatic morphology and function in DM rats, as indicated by improvements in survival markers, the inhibition of apoptotic signaling and the upregulation of the AMPK/Sirt1 axis [104]. The combined administration of RSV and MSCs led to the most marked improvement in DN via paracrine mechanisms or immune regulation [105]. Khalil et al. further noted that both MSCs preconditioned ex vivo with RSV (MCR) and MSCs isolated from rats pretreated with RSV (MTR) homed successfully to injured pancreata and showed therapeutic potential in the treatment of STZ-induced type 1 diabetes mellitus (T1DM) [106]. MTR and MCR restored pancreatic regenerative capabilities via activation of PI3K/Akt signaling while suppressing oxidative stress, apoptosis, and fibrosis. Notably, MCR cells were more effective than MTR cells.

In recent years, *grape seed* extract (GSE) has become increasingly popular as a nutritional supplement in many countries. GSE has been found to have significant antioxidant and immunomodulatory effects. As a stimulator and protector of MSCs, GSE dramatically manages the homeostasis of glucose and insulin secretion and improves the levels of inflammatory markers and oxidative stress [107]. The incubation of ADSCs with EGCG increased the cell viability under HG stress in a cell model via the expression of the survival marker p-Akt, which increased the paracrine effects of ADSCs to ameliorate pancreatic tissue damage. E-ADSCs (EGCG-pretreated ADSCs) showed significant improvements in several aspects, including decreased serum glucose levels; decreased serum and pancreatic oxidative stress; increased expression of the survival marker p-Bad; increased expression of the antioxidant marker Sirt1; suppressed expression of the apoptotic markers caspase 8, Bax, cytochrome C (Cyto-c), and caspase 3; and increased islet size [52]. Lawsone-preconditioned hUCMSCs showed better antihyperglycemic effects and β cell regeneration than did untreated hUCMSCs [108]. The nonpolar fraction (NPF) of *green beans* (*Phaseolus vulgaris L*.) is composed of isolated compounds, including alotroproceryl acetate, fridelin, calotroproceryl A, and stigmasterol, which increase the homing of MSCs to the pancreas, potentiating their antidiabetic activity [109]. Hassen et al. [110] investigated the role of BMSCs and/or MT in improving beta cell functions in diabetic rats with STZ-induced diabetes. Co-treatment with BMSCs and MT improved pancreatic tissue and reduced the number of damaged beta cells. It can be concluded that cotreatment with stem cells and MT plays a significant role in restoring the structural and functional efficiency of beta cells in the pancreas more than treatment with stem cells alone. These results may be due to the role of MT as an antioxidant in increasing the efficiency and viability of MSCs. Another study confirmed the potential role of MT-pretreated ADSCs in enhancing the treatment and regeneration of islet cells from Langerhans in STZ-induced DM rats. It could be concluded that pretreatment of ADSCs with MT enhances the protection of the cells of the pancreatic islets of Langerhans, which was demonstrated by increased body weight and serum insulin levels with a decrease in fasting blood glucose levels. Compared with those in the STZ group, a significant decrease in the proinflammatory cytokine IL-17 and an increase in the anti-inflammatory cytokine IL-10 were also detected. A significant increase in the proliferating cell nuclear antigen (PCNA) index and the homing and proliferation of MSCs are associated with a decrease in caspase-3 [111].

Type 2 diabetes mellitus (T2DM) mice were fed a high-fat diet (HFD) combined with low-dose STZ injection. ADSCs promoted the recovery of multiple organs and had a therapeutic effect on T2DM from the perspective of the overall organ microenvironment. In addition, MT further augments the therapeutic effect of ADSCs. MT was added to canine ADMSC culture medium, and the treated cells were used to treat T2DM. After cocultivation with MT, MT first combines with MT1/MT2 and then activates the TGF-β family, thereby affecting the cell cycle of ADSCs and promoting their viability [112]. The data demonstrated that MT-ADSCs restore hyperglycemia, insulin resistance, insulin sensitivity, and glucose metabolism by restoring inflammation and ER stress in the pancreas and liver in mice and dogs with T2DM. MT improved the anti-inflammatory and anti-ER stress abilities of ADMSCs through TGF-β and improved the therapeutic effect, which is safe and valuable for pet clinics.

#### Pancreatitis

Pancreatitis is an inflammatory disease of the pancreas that arises when pancreatic juice flow is impeded or refluxes, causing digestion of the pancreas and surrounding tissues by digestive enzymes. And pancreatitis is divided into severe acute pancreatitis (SAP) and chronic pancreatitis (CP). For CP, ICA combined with MSCs treatment can impressively improve the function of pancreatic stellate cells by enhancing the key β-cell markers PDX1 and MafA [113]. It restoredPDGF, monocyte chemoattractant protein 1 (MCP-1) and collagen-I to basal levels and modulated TGF-β, which suggests that it plays a vital role in the healing of fibrosis. Moreover, a significant decrease in the levels of the cytokines IL-8 and TNF-α, and a significant amelioration of myeloperoxidase activity were noted. In addition, a reduction in MCP-1 and collagen type-1 levels along with Hedgehog signaling downregulated the expression of Patched-1 (PTCH-1), Smoothened (SMO), and GLi-1 (a glioma-associated oncogene). Liu et al. [114] pointed that RSV pretreatment may be a new strategy to improve the therapeutic effect of BMSCs on SAP. RSV preprocessed BMSCs can activate the P13K/AKT signaling pathway, secrete vascular endothelial growth factor A (VEGFA) and inhibit pancreatic cell apoptosis, achieving the therapeutic effect of resisting apoptosis of pancreatic cells and promoting regeneration of damaged blood vessels.

### Other organ disease and injury

#### Chronic respiratory diseases

Trauma, air pollution, long-term smoking, an aging population and various respiratory virus infections (such as 2019 coronavirus disease (COVID-19)) have great negative impacts on lung health. Chronic respiratory diseases, including asthma, chronic obstructive pulmonary disease (COPD), idiopathic pulmonary fibrosis (IPF), pulmonary hypertension and occupational diseases, affect more than 500 million people worldwide [115].

COPD is a sustained blockage of the airways due to lung inflammation occurring with chronic bronchitis and/or emphysema. Chen et al. [116] generated HSP-VEGFA-MSCs by transducing mesenchymal stem cells using the lentiviral vector HSP70-VEGFA and reported that both cis-resveratrol (c-RSV) and trans-resveratrol (t-RSV) activated the HSP70 promoter in MSCs to induce VEGFA expression. Moreover, in an ex vivo aortic ring assay and a 3D scaffold in vivo model, c-RSV-treated MSCs promoted angiogenesis, while t-RSV-treated MSCs had fewer blood vessels. These authors further reported that genetically modified c-RSV-treated MSCs exhibited beneficial effects, such as upregulated expression of antioxidant-regulated nuclear factor erythroid 2-related factor (Nrf2), oxygenase 1 (HO-1) and manganese superoxide dismutase (MnSOD) genes, inhibition of inflammatory cytokines, replacement of dysfunctional endothelial cells, engraftment into the pulmonary wall and reactivation of lung regeneration signaling pathways [117]. These results support the hypothesis that the expression of VEGA induced by c-RSV acting on the HSP70 promoter in transplanted MSCs enhances the antigenic effects of stem cell gene therapy.

Allergic asthma is also a public health problem. It is a chronic inflammatory disease characterized by airflow obstruction and airway hyperresponsiveness. Airway remodeling is a symbolic feature of allergic asthma, but there is no effective clinical measure to reverse this phenomenon. Pretreatment of BMSCs with the omega-3 fatty acid eicosapentaenoic acid (EPA) reduced the inflammatory cell counts in Bronchoalveolar Lavage Fluid (BALF), the levels of TH2 cytokines (IL-4 and IL-13), and the occurrence of pulmonary fibrosis and mucinous cells and improved lung function in experimental allergic asthma induced by house dust mites (HDMs) [118]. Moreover, pretreatment of MSCs with EPA promoted the polarization of pulmonary macrophages, resulting in the anti-inflammatory effects of M2 macrophages.

#### Acute respiratory diseases

Acute respiratory distress syndrome (ARDS) is the most serious form of acute lung injury (ALI). ALI/ARDS is a respiratory disease caused by bacteria and viruses and severe chest trauma. It is characterized by abnormal lung structure, excessive inflammation, such as increased neutrophils, and edema caused by increased pulmonary vascular permeability. MSCs can effectively reduce alveolar permeability protein and reduce damage to the pulmonary endothelium and pulmonary epithelium [124]. In addition, EPA can improve the therapeutic effects of MSCs in experimental cecal hligation and puncture (CLP)-induced sepsis. Pretreatment of ADSCs with EPA can reduce lung inflammation, edema and alveolar collapse; reduce the levels of IL-1β, KC and TGF-β; increase the level of VEGF; and improve lung function [125]. In addition, pretreatment of ADSCs with EPA not only reduced lung tissue damage but also reduced distal organ tissue damage, which led to a decrease in the severity of sepsis and an improvement in the survival rate. The transplantation of hUCMSCs pretreated with pyrogallic acid improved and enhanced LPS-mediated lung pathological changes, lung index (lung/body ratio) increases, epithelial cell apoptosis, TLR4/NF-κB signal activation and proinflammatory cytokine release [126]. In addition, other studies have shown that small molecule-regulated MSCs can better repair lung diseases, as shown in Table 5 (Additional file 5). Pretreatment of ADSCs with MT can significantly increase arterial oxygen saturation and right ventricular systolic blood pressure and reduce alveolar injury and pulmonary interstitial edema after acute lung IR injury [127].

#### Inflammatory bowel diseases

The gastrointestinal (GI) tract is the largest immune organ in the human body and is responsible for the digestion and absorption of nutrients, the elimination of waste and the prevention of harmful pathogens. Diseases and injuries in the GI tract have direct effects on fitness and health. Astragaloside and baicalin have been shown to enhance cell survival, inhibit apoptosis, and regulate inflammation in LPS-induced MSCs in vitro. Consequently, more MSCs can be recruited to the injured site to support repair of the intestinal mucosa barrier [38]. SD rats were fed 3% (w/v) dextran sulfate sodium (DSS) in water for 10 days to induce experimental colitis. After DSS feeding, the rats developed typical symptoms of inflammatory colitis, including loss of weight, diarrhea, bloody stool, and death, which caused severe inflammation and damage to the colon mucosa. The administration of MT-pretreated BMSCs significantly alleviated DSS-induced body weight loss, diarrhea, bloody stool, colon inflammation and damage, which partially reversed the therapeutic dysfunction of P25 BMSCs [128]. These findings suggest that MT treatment enhances the immunomodulatory properties of long-term passaged MSCs. Recent studies have shown that identifying NSMs can counteract inflammatory damage in BMSCs, which is a pharmacological solution that can promote the immunosuppressive effects of BMSCs from the inflammatory microenvironment to improve colitis. Osthole, a natural pyroxanthin originally extracted from the *Cnidium* plant, is commonly utilized in traditional Chinese medicine. It has been shown to possess diverse functions, such as osteogenic, hepatoprotection, cardiovascular protection, anti-inflammatory, antitumor and antimicrobial activities. The administration of osthole-treated MSCs restored the immunosuppressive capacity of BMSCs, resulting in reduced lymphocyte infiltration and mucosal damage in experimental inflammatory colitis [37]. Moreover, the implantation of RSV-treated periodontal ligament-derived stem cells (PDLSCs) inhibited the infiltration of inflammatory T cells in mice with colitis and effectively restored epithelial structure, which was mediated by the activation of FASL induced by ERK [129]. Additionally, pretreatment of PDLSCs with 10% RSV had comparable effects on colitis treatment, suggesting that NSMs augment therapeutic efficacy and have the potential to significantly reduce the dosage of MSCs required for disease management.

## Conclusion and perspectives

**Fig. 2:**
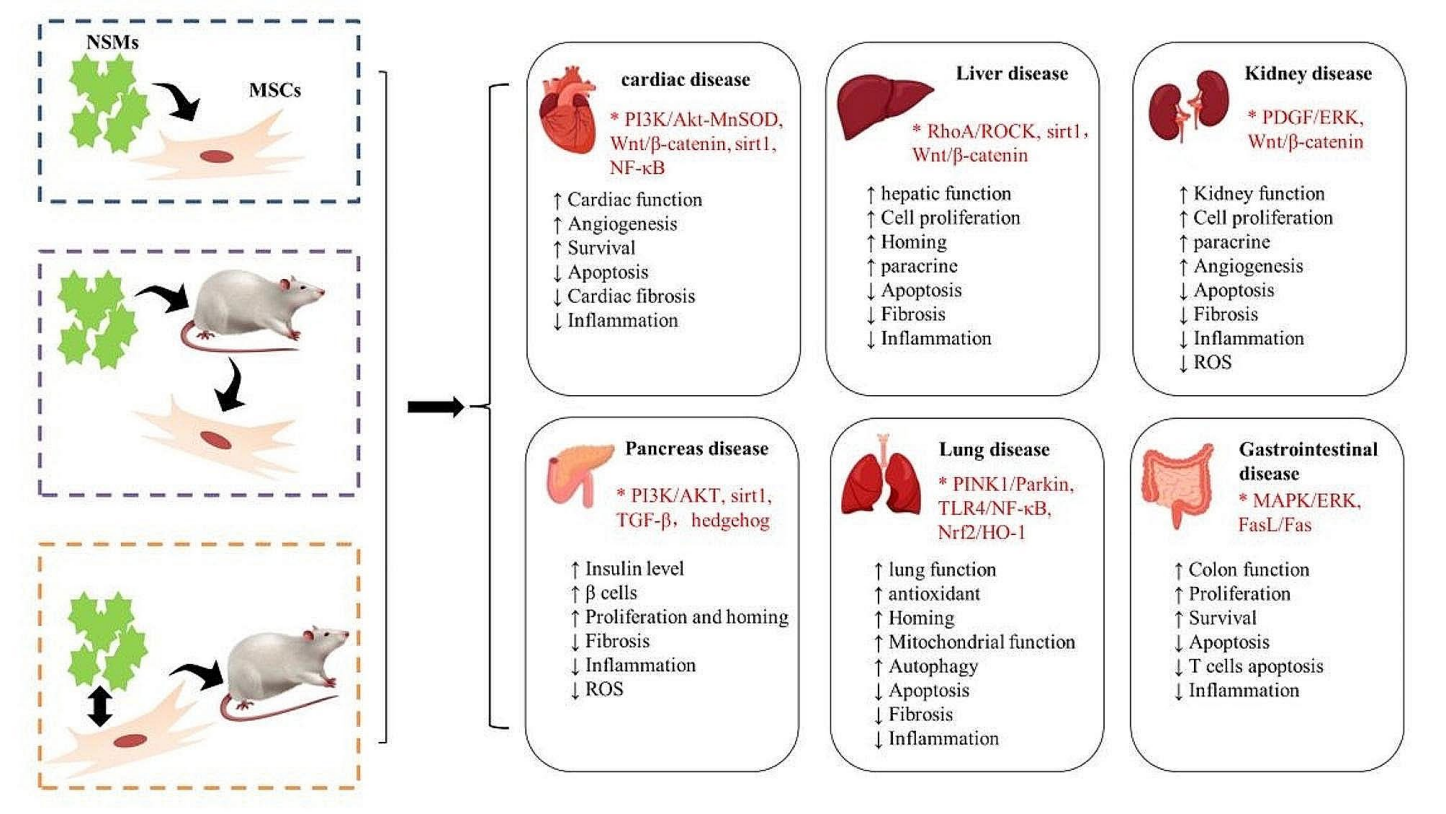
Schematic diagram of the tissue repair and regenerative functions of MSCs in internal organs under the action of NSMs. (Created with Microsoft PowerPoint 2010 and Adobe Photoshop CS4.)

In this review, MSCs preconditioned with NSMs or MSCs and NSMs infused simultaneously possess enhanced biological features and therapeutic potential for injury repair in vital organs, including the heart, liver, kidneys, pancreas, and other internal organs (Fig. 2). Although there are exciting discoveries thus far, the understanding of the regulation of MSCs by NSMs is still in its infancy. It has a number of bottlenecks that need to be addressed. First, increasing evidence has shown that MSCs from different sources retain certain organ-specific functions and properties, including gene expression and stability, cell-surface proteins, differentiation patterns, secreted cytokine profiles, and immunomodulatory ability [130, 131]. Therefore, these differences may have a great impact on the survival rate and therapeutic characteristics of MSCs [132], and further experimental studies should be carried out to compare and evaluate the effects of MSCs from different sources and to clarify whether cells from one source may have a greater therapeutic effect on the target disease than cells from other sources.

In addition, the combined use of NSMs and MSCs is diverse. For example, MSCs were isolated from NSMs-pretreated rats, MSCs were preconditioned with NSMs, or MSCs were infused with NSMs at the same time. The source of MSCs and the mode of action of NSMs are worthy of further study. Moreover, the dosage, intervals, frequency and method of MSCs infusion are not standardized, which limits research on the regulatory effects of NSMs on the ability of MSCs to promote tissue repair and regeneration.

Second, some NSMs, such as GSE and AOF, are derived from natural plant extracts and play an auxiliary role in regulating the function of MSCs as complex compounds. Further research is needed to identify and characterize the main active components of these extracts to better understand their therapeutic effects and mechanisms. At the same time, it is necessary to study the effects and mechanism of NSMs in more detail. There are still many defects in the reported regulatory effects of NSMs on MSCs in existing studies, such as insufficient sample size, incomplete experimental grouping, and insufficient mechanistic studies. Moreover, pretreatment of UCMSCs with asarinin significantly promotes the immunosuppressive effects of MSCs after HSCs transplantation [136], whereas it may have multiple cytotoxic effects, including arrhythmia, respiratory center depression, hepatotoxicity, and nephrotoxicity [137]. Therefore, more studies are needed to confirm the safety, effectiveness and mechanism of NSMs.

Third, additional large-scale clinical trials are required to evaluate their safety and efficacy in the human body. This would be helpful to provide a better understanding of their clinical applicability and potential for use in regenerative medicine.

Finally, even with the addition of NSMs, the rate and duration of MSCs engraftment are too limited to justify the remarkable results observed in tissue repair. In particular, it has been proposed that endogenous MSCs migrate from their perivascular location and create a regenerative microenvironment through the secretion of bioactive factors and the modulation of the immune cell response during tissue injury. Moreover, NSMs can mobilize endogenous MSCs to play a repairing role. RSV activated endogenous cardiac stem cells (CSCs), increased capillary density and decreased cardiomyocyte apoptosis in the ischemic myocardium, possibly through the upregulation of VEGF and stromal cell-derived factor 1 (SDF‑1α) [139]. Ke et al. found that CA can increase the MMP and adenosine triphosphate (ATP) production in lung mesenchymal stem cells (LMSCs), reduce ROS and the apoptosis rate, and reverse H_2_O_2_-induced damage to the mitochondrial structure of pulmonary interstitial cells [141]. In addition, CA could upregulate the PINK1/Parkin signaling pathway and activate autophagy to prevent IPF. They also found that curcumin (CUR) can decrease ROS secretion and increase MMP expression in LMSCs by regulating the Akt/Nrf2/HO-1 pathway after pretreatment of LMSCs to resist IPF [142].

Of note, there is much interest in which NSMs culminate in the use or/and modulation of the MSCs-derived secretome (EVs or conditioned medium (CM)), with multiple studies being performed for different types of disorders and medical conditions. Gehan et al. compared the efficacy of exosomes (Exos) obtained from CUR-preconditioned MSCs (MSCs/Exos-CUR) with that of Exos obtained from normal MSCs/Exos, and the results indicated that Exos derived from CUR-preconditioned MSCs were able to ameliorate and protect against the recurrence of NAFLD. Compared with MSCs/Exos treatment, MSCs/Exos-Cur treatment significantly improved steatosis and inflammation; decreased the serum levels of liver enzymes, liver triglycerides (TGs) and cholesterol (Ch); and increased lipid peroxidation in a mouse model [143]. AGS-IV-stimulated MSCs-Exos can improve myocardial contractile function, myocardial fibrosis and angiogenesis, reduce inflammatory factors and induce apoptosis in rats after AMI [144]. The effects of RG1-induced MSCs-Exos on myocardial function and fibrosis in DM mice were also evaluated. Exosomal circNOTCH1 secreted by RG1-induced MSCs can alleviate DCM by activating the NOTCH signaling pathway to induce M2 polarization of macrophages [145]. Radiation injury caused by radiotherapy triggers epithelial apoptosis or eventual necrosis in the intestine, which is also known as radiation-induced intestinal injury (RIII). Intraperitoneal injection of CM derived from RG-1 preactivated BMSCs (RG1-MSCs-CM) in irradiated rats significantly improved survival and promoted the structural and functional restoration of RIII by regulating intestinal regeneration, inflammation and angiogenesis. These results are mainly because RG1 stimulation significantly upregulates the expression of HO-1 and increases the secretion of the pivotal factors VEGF/IL-6 in BMSCs, thus promoting the recovery of RIII [146]. *Alhagi maurorum*, a semi shrub of the Fabaceae family, is a perennial herbaceous plant. In combination with MSCs extract, it can effectively inhibit the abnormal activation of the NOD-like receptor thermal protein domain associated protein 3 (NLRP3) inflammasome signaling pathway, reduce the expression of proinflammatory cytokines, and alleviate DSS-induced colon injury in mice with ulcerative colitis [147].

Future research should focus on further elucidating the underlying molecular mechanisms by employing advanced genomic, proteomic, and metabolic techniques. In addition, additional efforts should be made to investigate the potential synergistic effects of combining different NSMs on improving therapeutic outcomes. In brief, NSMs demonstrate great potential in the area of MSCs-based treatment for damage repair and regeneration of internal organs, and we hope that this review can provide a quick look and inspiration for research in relevant fields. By addressing these limitations and challenges and further exploring their mechanisms of action, these small molecules could revolutionize the field of regenerative medicine and contribute to the development of novel therapeutic approaches for treating organ damage and dysfunction.

## Data Availability

Not applicable.

## Cells

MSCs: Mesenchymal stem cells
NSMs: Natural small molecule compounds
HSCs: Hematopoietic stem cells
LSCs: Leukemic stem cells
iPSCs: Induced pluripotent stem cells
ADSCs: Adipose mesenchymal stem cells
BMSCs: Bone marrow mesenchymal stem cells
UCMSCs: Umbilical cord mesenchymal stem cells
rBMSCs: Rat BMSCs
aADSCs: Apoptotic ADSCs
AF-MSCs: Amniotic fluid-derived mesenchymal stem cells
WJMSCs: Wharton’s jelly derived MSCs
EC: Endothelial cell
LMSCs: Lung mesenchymal stem cells
CMCs: cardiomyocytes
CSCs: Cardiac stem cells
HSCs: Hematopoietic stem cells
PDLSCs: Periodontal ligament derived stem cells

## NSMs

APS: Astragalus polysaccharide
DN LA: Dendrobium nobile Lindl. Alkaloids
DPA: Dehydropachymic acid
PSP: Polygonatum sibiricum polysaccharide
ASP: Angelica sinensis polysaccharide
ABPS: Achyranthes bidentata polysaccharides
DOP: Dendrobium officinale polysaccharide
RSV: Resveratrol
EUG: Eugenol
BBR: Berberine
Tan IIA: Tanshinone IIA
AGS-IV: Astragaloside IV
AA: Ascorbic acid
Sal B: Salvianolic acid B
MT: Melatonin
RA: Retinoic acid
EGCG: Epigallocatechin-3-gallate
VitC: Vitamin C
GSE: Grape seed extract
EGb: Ginkgo biloba extract
RG1: Ginsenoside RG1
AOF: Alpinate Oxyphyllae Fructus
LPS: Lipopolysaccharide
Fa: Ferulic acid
VitE: Vitamin E
HA: Hyaluronic acid
EPA: Eicosapentaenoic acid
CA: Cinnamaldehyde
ICA: Icariin
CUR: Curcumin

## Diseases/Models

AML: Acute myeloblatsic leukemia
OVX: Ovariectomy
RIBE: X-ray radiation-induced bystander effects
CHDs: Coronary heart diseases
I/R: Ischemia/reperfusion
CVDs: Cardiovascular diseases
IHD: Chronic ischemic heart disease
AMI: Acute myocardial infarction
HF: Heart failure
DM: Diabetes mellitus
DCM: Diabetic cardiomyopathy
aGVHD: Acute graft-versus-host disease
NAFLD: Nonalcoholic fatty liver disease
SAP: severe acute pancreatitis
CP: Chronic pancreatitis
NASH: Nonalcoholic steatohepatitis
ALF: Acute liver failure
PH: Partial hepatectomy
AKD: Acute kidney disease
CKD: Chronic kidney disease
CP: Chronic pancreatitis
RIII: Radiation induced intestinal injury
IPF: Idiopathic pulmonary fibrosis
ARDS: Acute respiratory distress syndrome
ALI: Acute lung injury
DN: Diabetic nephropathy
T1DM: Type 1 diabetes mellitus
T2DM: Type 2 diabetes mellitus

## Others

ANKFY1: Ankyrin repeat and FYVE domain containing 1
PPARγ: Peroxisome proliferator activated receptor gamma
TRPV4: Transient Receptor Potential Vanilloid 4
Cx: Connexin
GJIC: Gap junctional intercellular communication
Cyto-C: Cytochrome C
AIF: Apoptosis inducing factor
VEGF: Vascular endothelial growth factor
Ang-1: Angiopoietin 1
VEGFA: Vascular endothelial growth factor A
PI3 K: Phosphoinositide 3-kinase
AKT: Protein kinase B
Bcl-2: B-cell lymphoma-2
XPA: Xeroderma pigmentosum group A
XPC: Xeroderma pigmentosum group C
ERCC1: Excision repair cross-complementation group 1
RPA1: Replication protein A1
RPA2: Replication protein A2
V-ATPases: Vacuolar H+-ATPases
FAC: Ferric ammonium citrate
NER: Nucleotide excision repair
FA: Formaldehyde
BMP-2: Bone morphogenetic protein-2
ROS: Reactive oxygen species
CXCR4: CXC chemokine receptor4
TGF-β: Transforming growth factor β
HGF: Hepatocyte growth factor
TNF-α: Tumor necrosis factor –alpha
IL: Interleukin
DOX: Doxorubicin
HE: Hematoxylin and eosin
UUO: Unilateral ureteral obstruction
HG: High-glucose
NPF: Nonpolar fraction
HFD: High-fat diet
PDGF: Platelet-derived growth factor
ERK: Extracellular signal-regulated kinase
MCP-: 1Chemoattractant protein 1
PCNA: Proliferating cell nuclear antigen
PTCH-1: Patched-1
SMO: Smoothened
GLi-1: Glioma-associated oncogene
COPD: Chronic obstructive pulmonary disease
BALF: Bronchoalveolar lavage fluid
CLP: Cecal hligation and puncture
Nrf2: Nuclear factor erythroid 2-related factor
HO-1: Oxygenase 1
MnSOD: Manganese superoxide dismutase
HDM: House dust mite
GI: Gastrointestinal
DSS: Dextran sulfate sodium
MMP: Mitochondrial membrane potential
SDF-1α: Stromal cell-derived factor 1
ATP: Adenosine triphosphate
Exo: Exosome
TG: Triglycerides
Ch: Cholesterol
CM: Conditioned medium
3D: Three dimensional
NLRP3: NOD-like receptor thermal protein domain associated protein 3
